# Identifying Preferred Learning Approaches For Dementia Training Of Direct Care Workers

**DOI:** 10.1093/geroni/igaf122.3608

**Published:** 2025-12-31

**Authors:** Troy Andersen, Daniel Kaplan, Norman Foster, Jeanine Stefanucci, Andrea Dremann, Chad Moffitt, Bonnie Bechek

**Affiliations:** University of Utah, Salt Lake City, Utah, United States; Adelphi University, Garden City, New York, United States; ProActive Memory Services, Inc., Proactive Memory Services, Utah, United States; University of Utah, Salt Lake City, Utah, United States; ProActive Memory Services, Inc., Salt Lake City, Utah, United States; University of Utah, Salt Lake City, Utah, United States; ProActive Memory Services, Inc., Salt Lake City, Utah, United States

## Abstract

Training direct care providers to care for individuals with dementia is challenging. Cultural differences, varying education levels, and high staff turnover further complicate consistent training. In our previous studies, direct care providers emphasized the need for training that is relevant, flexible and interactive. Our project aims to improve dementia care competencies through an innovative, flexible web-based platform that presents content in written and audio narration formats. We interviewed and tracked server data to identify preferences of 23 direct care providers (16 PCA, 7 ALC, 95.65% women, mean age 37.5) from personal care agencies (PCA) and assisted living communities (ALC) using training software an average of 1 hour per week for one month. Their use of audio versus written materials was tracked and analyzed to better understand learning preferences. All participants read the materials and 82% listened to the audio. We also captured the time windows of when the training was access to determine if integration of learning occurs within or outside of the work schedule. Highlights from the findings include 61% of participants accessed the training during traditional work hours, while 39% engaged the training outside of a traditional work schedule. This finding speaks to the challenges of integrating learning opportunities into the traditional work schedule. Direct care providers have diverse educational background, experience, and time constraints. To best meet their needs, content should be brief and presented in multiple formats for trainees to select as they believe will best fit their needs.

